# Dying from cancer or other chronic diseases in the Netherlands: ten-year trends derived from death certificate data

**DOI:** 10.1186/1472-684X-8-4

**Published:** 2009-02-04

**Authors:** Lud FJ van der Velden, Anneke L Francke, Lammert Hingstman, Dick L Willems

**Affiliations:** 1 NIVEL, Netherlands Institute for Health Services Research, Otterstraat 118-124, 3513 CR Utrecht, the Netherlands; 2 Department of general practice, AMC/UvA, Meibergdreef 15, 1105 AZ Amsterdam, the Netherlands

## Abstract

**Background:**

For the further development of palliative care, it is relevant to gain insight into trends in non-acute mortality. The aim of this article is twofold: (a) to provide insight into ten-year trends in the characteristics of patients who died from cancer or other chronic diseases in the Netherlands; (b) to show how national death statistics, derived from physicians' death certificates, can be used in this type of investigations.

**Methods:**

Secondary analysis of data from 1996 to 2006 on the "primary" or "underlying" cause of death from official death certificates filled out by physicians and additional data from 2003 to 2006 on the place of death from these certificates.

**Results:**

Of the 135,000 people who died in the Netherlands in 2006, 77,000 (or 57%) died from a chronic disease. Cancer was the most frequent cause of death (40,000). Stroke accounted for 10,000 deaths, dementia for 8,000 deaths and COPD and heart failure each accounted for 6,000 deaths. Compared to 1996, the number of people who died from chronic diseases has risen by 6%.

Of all non-acute deaths, almost three quarters were at least 70 years old when they died. Almost one third of the people died at home (31%), 28% in a hospital, 25% in a nursing home and 16% somewhere else.

**Conclusion:**

Further investments to facilitate dying at home are desirable. Death certificate data proved to be useful to describe and monitor trends in non-acute deaths. Advantages of the use of death certificate data concern the reliability of the data, the opportunities for selection on the basis of the ICD-10, and the availability and low cost price of the data.

## Background

Some people die acutely and unexpectedly. Others die from a chronic disease. In the latter group death occurs after a more or less long period of sickness. They are therefore likely to experience palliative care needs. For the planning and organisation of palliative care it is important to gain insight into the background characteristics of people who die from cancer or other chronic diseases. This is especially important in the context of an ageing population, which will ultimately lead to a greater number of deaths.

In the year 2000 the authors performed a first analysis of non-acute death. [1] The analysis of non-acute death presented here is an update and an extension of the study performed in 2000. This update was necessary because the previous study concerned mortality data that are now ten years old. It was also important because on a national and international level palliative care is seen as a spearhead of healthcare policy. [2,3]

Research based on death certificate data may be used as an information source and monitoring tool for healthcare policy. Although used in some studies in the USA, [4-11] and a selection of European countries, [12-17] the use of death certificate data for scientific research remains rather underexploited.

The aim of this article is twofold:

(a) to provide insight into ten-year trends regarding characteristics of patients who died from cancer or other chronic diseases in the Netherlands;

(b) to show how national death statistics, derived from physicians' death certificates, can be used in this type of investigation.

This article will address the following questions about non-acute death:

* How many people in the Netherlands died from cancer or other chronic diseases in 2006?

* What are their demographic characteristics (age group and gender)?

* How has the number of people who died from cancer or other chronic diseases changed in the Netherlands between 1996 and 2006?

* What was the place of death (e.g. at home, in a hospital) of people who died from a chronic disease in 2006 and has this fact changed in the past few years?

We will also address a more contemplative and methodological question, namely:

* What are the opportunities and limitations of using death certificate data for describing trends in non-acute mortality?

## Methods

Data about the total number of people who died in the Netherlands from a chronic disease between 1996 and 2006, including their primary cause of death, age and gender, were obtained from Statistics Netherlands (in Dutch: Centraal Bureau voor de Statistiek, or, abbreviated, the CBS). These data are based on official death certificates. For the period 2003–2006, also the place of death could be obtained.

The Netherlands, as other western countries, has an obligatory death reporting system in which the physician who treated the deceased at the time of death or a pathologist fills out the death certificate. When filling out this certificate the physician has to follow the cause-of-death certification guidelines of the World Health Organisation (WHO). Physicians are asked to give up to 4 causes of death, with one of them being the "primary" or "underlying" cause that started the chain of events leading to death. Effects of complications of the disease that is considered as the "primary" cause are called "secondary" causes.

All causes mentioned by the physician are coded by coders at the CBS in accordance with the WHO coding guidelines. The 10-th edition of the International Classification of Diseases (ICD-10) is used to code the causes of death from 1996 onwards.

Statistics on deceased people are regularly published by Statistics Netherlands, with in general a one year time lag. Only the data on the primary cause are readily available. Since a few years, the Statline website is the most important medium for publication . In addition, Statistics Netherlands offers researchers the opportunity to do scientific research – under strict conditions, related to protecting the anonymity of the deceased individuals – by using the data in their databases.

We gathered several aggregated tables with number of deaths broken distinguished by year of death (1996–2006), gender (Male-Female), age at death (5- or 10-year age groups), cause of death (2- or 3-digit ICD-10 codes) and, for 2003–2006, place of death (hospital, nursing home, home for the elderly, home or other). Most of these tables were downloaded from Statline, but the tables with place of death were delivered on demand by Statistics Netherlands.

The aggregated tables contained no data that could be reduced to individuals. Therefore, according to Dutch law ethical approval for this study was not needed.

Within the total set of death certificate data we selected data from people with a chronic disease as primary cause of death (table 1). The selected diseases represent the main chronic diseases, with at least 50 people who have died from such a specific chronic condition in the Netherlands. Chronic diseases from which only very few people had died, such as asbestosis or cystic fibrosis, were not included.

**Table 1:** Selected primary causes of death

**Diagnosis**	**ICD-10 code**
Cancer (including neoplasms with uncertain behaviour)	C00–C97 and D37–D48
Cerebrovascular diseases (stroke)	I60–I69
Dementia	F00–F03 and G30
Chronic obstructive pulmonary disorders (COPD)	J40–J47
Heart failure	I50
Diabetes	E10–E14
Parkinson's disease	G20–G21
Chronic kidney disease	N03–N04, N11–N13 and N18
Chronic liver disease	K70 and K72–K74
Spinal muscular atrophy and related disorders	G12
Multiple sclerosis (MS)	G35
Neuromuscular disorders	G70–G71
Acquired immunodeficiency syndrome (AIDS)	B20–B24

For this article, we used only some basic descriptive statistical techniques to analyse the data.

## Results

### Number of deceased people in 2006 by cause of death

**Figure 1:**
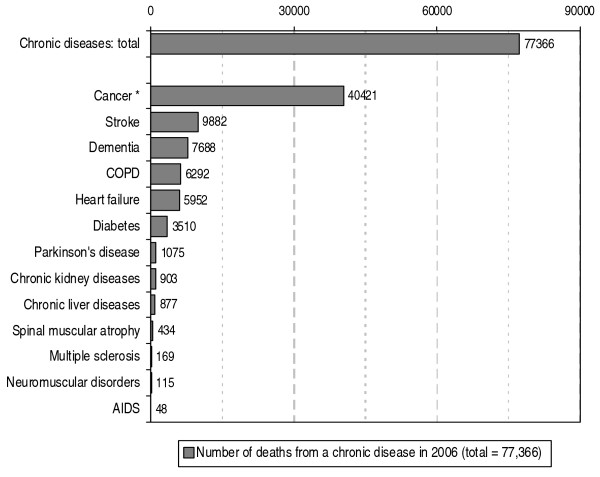
**Number of deaths in the Netherlands from chronic diseases in 2006 according to primary cause of death**. Source: CBS (deaths); processed by NIVEL (selection of chronic diseases, a.o.). *: Cancer, including death from neoplasms with uncertain behaviour.

In 2006 approximately 77,000 people in the Netherlands died from one of the chronic diseases we selected. This number accounts for 57% of the total number of 135,000 people who died that year. In a little over 40,000 deceased people cancer was the primary cause of death (see figure 1). The number of people who died from any of the other selected chronic diseases was considerably lower. Stroke accounted for 10,000 deaths, dementia for 8,000 deaths and COPD and heart failure each for 6,000 deaths.

### Death by age and gender

**Figure 2:**
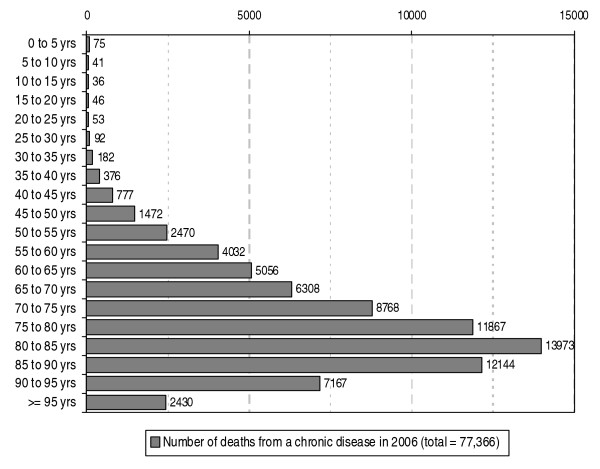
**Number of deaths in the Netherlands from chronic diseases in 2006 according to age**. Source: CBS (deaths); processed by NIVEL (selection of chronic diseases, a.o.)

The age distribution of the people who died from a chronic disease in 2006 shows a peak in the 80–85 years of age group (see figure 2) and almost three quarters (namely 73%) is at least 70 years old. The spread among the age groups does vary somewhat depending on the chronic disease (not shown here). For example, in the case of people who died from cancer a peak is seen in the 75–80 years of age group. For stroke, COPD, diabetes, Parkinson's disease and kidney diseases a peak is seen in the 80–85 years of age group and for dementia and heart failure in the 85–90 years of age group.

**Figure 3:**
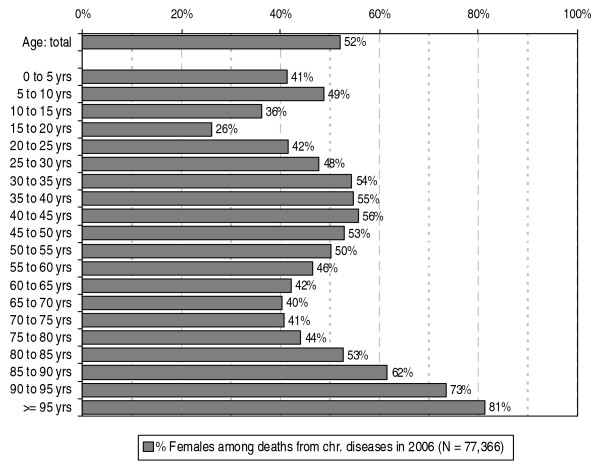
**Percentage females among deaths from chronic diseases in the Netherlands in 2006 according to age**. Source: CBS (deaths); processed by NIVEL (selection of chronic diseases, a.o.)

Of all the people who died from a chronic disease in 2006, 52% were women (see figure 3). The proportion of women appears to vary with age: in the 30–50 years of age group the proportion of women within the people who die from a chronic disease is consistently around 55%. From the age of 50 the proportion of women decreases to about 40% for the 65–70 and 70–75 years of age groups and then increases to 81% for the highest age group.

### Developments in non-acute deaths between 1996 and 2006 by cause of death

**Figure 4:**
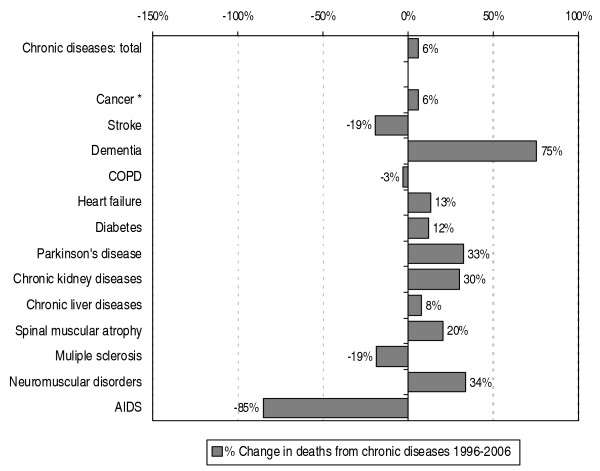
**Percentage change in deaths from chronic diseases in the Netherlands from 1996–2006 according to primary cause of death**. Source: CBS (deaths); processed by NIVEL (selection of chronic diseases, a.o.). *: Cancer, including death from neoplasms with uncertain behaviour

The number of non-acute deaths has increased with 6% between 1996 and 2006 (see figure 4). In case of cancer the increase also turns out to be 6%. The largest increase in number of deaths was seen in the diagnosis category dementia: 75%. The largest decrease was seen in the category AIDS, namely a decrease of 85%. See figure 4 for the developments in mortality related to other chronic diseases.

The number of non-acute deaths from 1996 to 2006 by cause of death are presented for each year in table 2. For the total number of non-acute deaths there were a number of years with at least a 0.9% increase: 1998, 1999, 2001 and 2002. From 2002 onwards, the total number of deaths grew only by less than 0.9%, and in 2004 there was even a decline of -1.7%. For each of the different chronic diseases, different trends or changes in trends are visible. For cancer, the number of deaths slightly increased almost each year with about 1%, but showed a small decrease in 1997 and 2000. For stroke, the number of deaths decreased in most years with -1% up to -6% per year, with the exception of 1999 and 2002.

**Table 2:** Number of deaths from chronic diseases in the Netherlands from 1996–2006 according to primary cause of death and percentage change relative to the preceding year

**Diagnosis**	**1996**	**1997**	**1998**	**1999**	**2000**	**2001**	**2002**	**2003**	**2004**	**2005**	**2006**
Cancer *	38,157	37,968 -0.5%	38,266 0.8%	39,005 1.9%	38,702 -0.8%	38,917 0.6%	39,579 1.7%	39,789 0.5%	40,196 1.0%	40,252 0.1%	40,421 0.4%
Stroke	12,232	12,146 -0.7%	12,104 -0.3%	12,409 2.5%	12,184 -1.8%	11,890 -2.4%	12,222 2.8%	11,469 -6.2%	10,990 -4.2%	10,326 -6.0%	9,882 -4.3%
Dementia	4,383	4,588 4.7%	4,653 1.4%	5,203 11.8%	5,343 2.7%	5,827 9.1%	6,840 17.4%	7,046 3.0%	6,990 -0.8%	7,005 0.2%	7,688 9.8%
COPD	6,488	6,356 -2.0%	6,974 9.7%	6,740 -3.4%	6,753 0.2%	6,373 -5.6%	6,310 -1.0%	6,548 3.8%	5,755 -12.1%	6,423 11.6%	6,292 -2.0%
Heart failure	5,272	5,265 -0.1%	5,322 1.1%	5,551 4.3%	5,908 6.4%	5,530 -6.4%	5,598 1.2%	5,830 4.1%	5,624 -3.5%	5,943 5.7%	5,952 0.2%
Diabetes	3,143	3,198 1.7%	3,224 0.8%	3,308 2.6%	3,345 1.1%	4,283 28.0%	4,079 -4.8%	3,791 -7.1%	3,769 -0.6%	3,759 -0.3%	3,510 -6.6%
Other chr. diseases	3,304	3,338 1.0%	3,259 -2.4%	3,395 4.2%	3,256 -4.1%	3,337 2.5%	3,444 3.2%	3,630 5.4%	3,450 -5.0%	3,480 0.9%	3,621 4.1%
**Total**	**72,979**	**72,859** **-0.2%**	**73,802** **1.3%**	**75,611** **2.5%**	**75,491** **-0.2%**	**76,157** **0.9%**	**78,072** **2.5%**	**78,103** **0.0%**	**76,774** **-1.7%**	**77,188** **0.5%**	**77,366** **0.2%**

Source: CBS (deaths); processed by NIVEL (selection of chronic diseases, a.o.)

*: Cancer, including death from neoplasms with uncertain behaviour

### Place of death in 2006 according to age and gender

**Figure 5:**
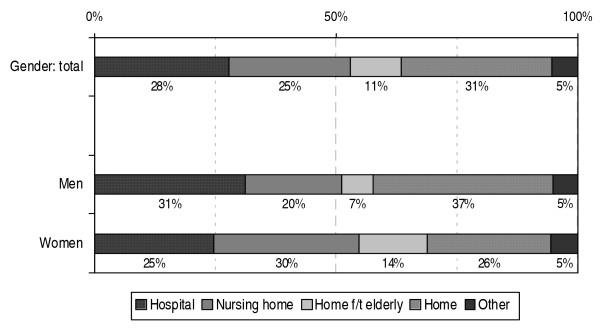
**Place of death for people who died from a chronic disease in the Netherlands in 2006 according to gender**. Source: CBS (deaths); processed by NIVEL (selection of chronic diseases, a.o.)

In 2006, approximately 28% of deaths related to cancer or other chronic diseases in the Netherlands occurred in a hospital, 25% in a nursing home and 11% in a home for the elderly (see figure 5). The proportion of patients who died at home was 31%. In 5% the place of death was either another place than those mentioned before or unknown. It may be assumed that 'other' includes, for instance, non-acute deaths in institutions for the mentally handicapped, mental healthcare institutions or one the 230 independent hospices that exits nowadays in the Netherlands. Ten years ago, the number of independent hospices was only some 40.

The proportion of men who died from a chronic disease in a hospital is somewhat higher than the proportion of women (31% versus 25%; see figure 5). The proportion of men who died from a chronic disease in a nursing home or home for the elderly, however, is much smaller than the proportion of women: 20% and 7% compared to 30% and 14%, respectively. Thirty-seven percent of the male patients died at home compared to 26% of the female patients.

The chance of dying at home was highest for people who were not yet very old: as high as approximately 50% of the non-acute deaths of people up to 70 years of age occurred at home (not show here). For the non-acute deaths of people 70 years of age and older the proportion who died at home gradually decreased with age. At comparable ages fewer women than men died at home.

### Developments in place of death

**Figure 6:**
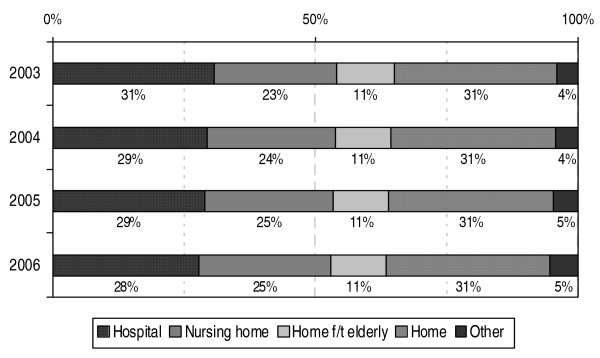
**Place of death for people who died from a chronic disease in the Netherlands in 2003–2006**. Source: CBS (deaths); processed by NIVEL (selection of chronic diseases, a.o.)

As Statistics Netherlands did not collect data on place of death until 2003, developments in the place of death in case of non-acute deaths are only analysed for the period 2003–2006 (see figure 6). The proportion of people who died in a hospital from one of the specified chronic diseases appears to have decreased somewhat in the past four years, from 31% in 2003 to 28% in 2006. The proportion of people who died in a nursing home has, at the same time, increased somewhat, from 23% in 2003 to 25% in 2006.

## Discussion

### Cause of death

In 2006, a total of 77,000 people in the Netherlands died from cancer or one of the other specified chronic diseases (stroke, dementia, COPD, heart failure, diabetes, Parkinson's disease, chronic kidney diseases, chronic liver diseases, spinal muscular atrophy, MS, neuromuscular disorders and AIDS). This group accounts for 57% of all the deaths in the Netherlands. The number of 77,000 may be a small underestimation of non-acute deaths as smaller diagnosis groups have not been included in our analyses. But it could also be an overestimate, because some people who had a chronic disease as primary cause of death, could in fact have died of acute complications of these chronic diseases.

In a study by Van der Heide et al. [18] the authors arrive at a much higher percentage of non-acute deaths in the Netherlands: these authors observe, on the basis of interviews with physicians, that almost 70% of deaths in the Netherlands in 2001/2002 was non-acute. The discrepancy between the findings of Van der Heide et al. and our analyses are probably connected with differences in research systematic: we based ourselves on data of death certificates filled out by physicians, whereas Van der Heide et al. based themselves on the non-acute deaths as indicated by the physicians in a survey. Particularly in very old people whose condition gradually deteriorates death is almost never entirely unexpected and acute, even though most patients will probably die from another cause than one of our specified chronic diseases. This indicates that the way in which non-acute deaths have been operationalised and assessed needs to be taken into account when interpreting the sometimes divergent non-acute death percentages.

### Selection of diseases

The way in which we operationalised the term 'non-acute deaths', is open for discussion. We tried to select all deaths with a chronic disease as 'primary' or 'underlying' cause. Hereby we also included people who died from an acute complication of a chronic disease as an underlying cause. We intend to repeat our study using multiple causes, which are divided between primary and secondary causes. Then we can make a distinction between people dying from acute or chronic complications of chronic diseases.

We also had a somewhat arbitrary exclusion of people who died from some chronic diseases. Although most of these excluded chronic diseases are quite small, some categories account for a substantial amount of up to several hundreds or more. For chronic kidney diseases one could think of including N05 (unspecified nephritic syndrome) or N19 (unspecified renal failure). These would add another 10 respectively 327 deaths to the 903 deaths from chronic kidney diseases mentioned in this article for 2006. We also could have included all "sequelae" (or "late consequence") codes as a non-acute cause of death, like B90 (Sequelae of tuberculosis, with 45 deaths), G09 (Sequelae of inflammatory diseases of the central nervous system, with 5 deaths) or I69 (Sequelae of cerebrovascular disease, with 522 deaths).

In the intended repetition of this study, we aim to look at heart diseases more thoroughly. Chronic heart diseases, like I25 (chronic ischemic heart diseases) result both in acute and chronic complications amenable to palliative care.

In a Dutch publication on this study [19] we decided to include for stroke only 67% of the deaths to account for 33% acute deaths. This was a very crude estimate and it could be argued that we overestimated the acute deaths for stroke in that publication. In the current article we included 100% of all stroke deaths. However, a certain part of the stroke deaths will be acute and unexpected. The acute inhospital mortality rate is 7% for ischemic stroke, according to Lee et al. [20] But for subarachnoid hemorrhage stroke they found 32% of acute inhospital mortality.

### Need for palliative care

In this study we looked at the number of non-acute deaths. However, the number of people who have a need for palliative care maybe somewhat lower. But we do not know how much lower. And we did not pay any attention to the amount or content of the palliative care that people used or are looking for.

### Place of death

Insight into the place of death is important, among other things, because it is increasingly regarded as an important quality indicator for good palliative care. [15] Experts in the field of palliative care often assume that dying at home is 'natural', that it enables people to keep control over their own life as long as possible and that it enhances the wellbeing of the patient and his family. [21-23] And it is at least equally important that patients themselves often have a clear preference for dying at home. [24,25] In light of this it is remarkable that only about one third of non-acute deaths in the Netherlands occur at home and more than a quarter in the hospital. According to a recent study by Cohen et al. the percentage of people who die at home in the neighbouring country Belgium is even somewhat lower and the percentage who die in a hospital somewhat higher than in the Netherlands. [15] For England and Wales, Gomes and Higginson showed that of all people who died in 2003, only 18% died at home. [17] They could also show that the percentage of people who die at home had declined from 1974 onwards. Our time frame was too small to show trends.

When someone dies in hospital it is very likely that some healthcare transitions have taken place in the final stages. Such transitions may bring about unrest and discomfort for both the patient and his family. Further investments from policy makers and healthcare providers to facilitate dying at home – such as good possibilities for consultation by general practitioners and home healthcare nurses and sufficient practical and emotional support for family members – are desirable.

### Use of death certificate data

This study also demonstrates that it is feasible to conduct cross-years research on the place of death and other socio-demographic characteristics of deceased people on a national level.

A big advantage of using death certificate data is that these data are relatively reliable: the causes of death mentioned in this article are filled out on the death certificate by the treating physician, not by someone who did not know the person who died.

Another advantage is that it is relatively easy to make reliable selections in causes of death, partly due to the use of the ICD-10. Other advantages are that the data are population based, relatively easy to obtain and at relatively little cost.

The usability of death certificate data to analyse trends in mortality on a national level has not only been established in our study, but for instance also in a study regarding ten-year trends in the place of death of cancer patients in England by Higginson et al. [13] From research by Cohen et al. [14-16], among others, it is known that death certificate data may also be useful to conduct cross-national research on mortality characteristics.

Despite their proven usability, the use of death certificate data also has its limitations. It is impossible, for instance, to be absolutely certain whether the cause of death as stated on the death certificate is in fact the real cause of death, because questions can remain considering the underlying causes, even after an autopsy. We are also not absolutely certain whether the death certificate data completely correspond with the data in medical records. [26,27] But since the death certificates are filled out by the treating physicians in case of cancer and other chronic diseases, the data on the death certificates and in the medical records will probably overlap to a large extent.

An important difference between our study and previous studies using death certificate data concerns the population. Previous research often focused on either a very specific population, such as persons who died from cancer, [4,12,13] or on the total deceased population. [5-8,10,11,14,15] Yet we based our study on a selection of people who had died from causes that are almost always preceded by a period of sickness and thus with a potential need for palliative care. As the ICD-10 is always used when death certificates are coded, it is relatively easy to make selections and thus to focus on the groups of patients that are most relevant within the scope of the planning and organisation of palliative care. It is interesting to find out in future research in other countries but in the same selection of causes of death, whether different or similar trends can be observed. Combining cross-years and cross-national comparisons of place of death and other socio-demographic data of people who have died from chronic diseases can be helpful to plan and monitor national and international policies with regard to palliative care.

## Conclusion

Further investments to facilitate dying at home are desirable. Death certificate data proved to be useful to describe and monitor trends in non-acute deaths. Advantages of the use of death certificate data concern the reliability of the data, the opportunities for selection on the basis of the ICD-10, and the availability and low cost price of the data.

## Competing interests

The authors declare that they have no competing interests.

## Authors' contributions

LvdV carried out the analysis and drafted the manuscript. AF conceived of the study and helped draft the manuscript. LH participated in its coordination and helped draft the manuscript. DW was responsible for the selection of "chronic diseases" and helped draft the manuscript. All authors read and approved the final manuscript.

## Pre-publication history

The pre-publication history for this paper can be accessed here:


